# The Effect of Early Rounds of *ex vivo* Expansion and Cryopreservation on the Adipogenic Differentiation Capacity of Adipose-Derived Stromal/Stem Cells

**DOI:** 10.1038/s41598-019-52086-9

**Published:** 2019-11-04

**Authors:** C. Durandt, C. Dessels, C. da Silva, C. Murdoch, M. S. Pepper

**Affiliations:** Institute for Cellular and Molecular Medicine, Department of Immunology, and SAMRC Extramural Unit for Stem Cell Research and Therapy, Faculty of Health Sciences, University of Pretoria, Pretoria, South Africa

**Keywords:** Mesenchymal stem cells, Stem-cell differentiation

## Abstract

Multipotent adipose-derived stromal/stem cells (ASCs) are candidates for use in cellular therapies for the treatment of a variety of conditions/diseases. *Ex vivo* expansion of freshly isolated ASCs may be necessary prior to clinical application to ensure that clinically relevant cell numbers are administered during treatment. In addition, cryopreserving cells at early passages allows for storage of freshly isolated cells for extended periods of time before expanding these cells for clinical usage. There are however several concerns that these laboratory-based procedures may alter the characteristics of the cells and in so doing decrease their regenerative potential. In this study we report on the impact of early rounds of cryopreservation (P0) and *ex vivo* expansion (P0 to P5) on the phenotypic characteristics and adipogenic differentiation potential of ASCs. Our results show that ASCs that upregulate CD36 expression during adipogenic differentiation gradually decrease with increasing expansion rounds. The consequent decrease in adipogenic differentiation capacity was evident in both gene expression and flow cytometry-based phenotypic studies. Successive rounds of expansion did not however alter cell surface marker expression of the cells. We also show that early cryopreservation of ASCs (at P0) does not affect the adipogenic differentiation potential of the cells.

## Introduction

The multipotency and self-renewal capabilities of mesenchymal stromal/stem cells (MSCs) make them attractive for application in tissue engineering and regenerative medicine^1–4^. Mesenchymal stromal/stem cells may be isolated from various tissues, including bone marrow, adipose tissue, umbilical cord and others^5^. Adipose tissue is a particularly attractive source as it contains a relatively high number of multipotent MSCs compared to other sources, and is generally associated with fewer ethical concerns^3,6,7^. The term “adipose-derived stromal/stem cell” (ASCs) refers to cells with multipotent capacity that are present within the stromal vascular fraction (SVF) obtained from adipose tissue^8^. Stromal vascular fraction (SVF) refers to the heterogeneous cellular fraction, excluding mature adipocytes, found within adipose tissue, and consists of ASCs, pre-adipocytes, endothelial cells, endothelial progenitor cells, smooth muscle cells, pericytes, fibroblasts, macrophages and others^9,10^.

Most clinical trials currently underway make use of SVF, with only a few studies using *ex vivo* expanded ASCs^11–14^. The predominant use of SVF in clinical trials is largely based on the Food and Drug Administration (FDA)’s view that cells cultured *ex vivo* are more-than-minimally manipulated cellular products, even if the cells are only cultured overnight^7,15,16^. However, the advantage of *ex vivo* expansion is that it will ensure that clinically relevant cell numbers can be achieved prior to initiation of treatment^4,17^. *Ex vivo* expansion also allows for the use of cells from a single donor in a clinical trial setting, and in so doing overcomes the challenges associated with inter-donor variability^18,19^. Developing allogeneic off-the-shelf cell therapy products in the future, that are ready for use at short notice, will also require the ability to expand cells *ex vivo* without compromising their regenerative properties^19^. However, it is still unclear to what extent *in vitro/ex vivo* manipulation impacts on the function, especially the regenerative properties, of ASCs. Several studies have indicated that MSCs, including ASCs, undergo fundamental changes during *ex vivo* expansion^16,20,21^. These *ex vivo*-associated changes include reduced proliferation potential, increased cell senescence, impaired multipotent differentiation capabilities and genomic instability^22^. The physiological relevance of some of these changes is poorly understood, contributing to the uncertainty regarding their use in the clinical setting. A better understanding of the impact that common laboratory practices, such as *ex vivo* expansion and cryopreservation, have on ASC function, will ensure that ASCs maintain their therapeutic potential after *ex vivo* manipulation when used clinically.

Acknowledged to be multipotent, MSCs have enhanced potential to differentiate into cells that comprise their tissue of origin^23,24^. In addition, the primary physiological function of ASCs is to differentiate into adipocytes^25^. Increased intracellular lipid accumulation is a key morphologic characteristic associated with adipogenic differentiation, and is regulated by a well-defined cascade of transcription factors. CCAAT/enhancer binding protein α (C/EBPα) and peroxisome proliferator-activated receptor γ (PPARγ) are main regulators^26–28^, with PPARγ being an essential master regulator of the adipogenic differentiation process^27^. Upon activation, these transcription factors induce the upregulation of enzymes responsible for fatty acid biosynthesis, transport and incorporation into triglycerides, the main component of intracellular lipid droplet cores^28^. Proteins that play an important role in fatty acid uptake include CD36 (a fatty acid translocase), fatty acid binding protein 4 (FABP4), and others^28^. Adipose-derived stromal cells constitutively express low levels of CD36 on their surface, with a sub-population that expresses higher levels of CD36^29,30^. Interestingly, CD36 is one of a few cell surface proteins that can be used to distinguish between ASCs and bone marrow-derived MSCs^31^.

We investigated the impact of early rounds of *ex vivo* expansion (P0 to P5) as well as initial cryopreservation following isolation (at P0) on the phenotypic characteristic and *ex vivo* adipogenic differentiation potential of ASCs. We found that a sub-population of ASCs with the ability to upregulate CD36 expression during adipogenic differentiation gradually decreases with increasing expansion rounds. The decrease in adipogenic differentiation potential of ASCs is significant from as early as P2. Cryopreservation at P0, however, did not affect the adipogenic differentiation potential of ASCs.

## Materials and Methods

### Materials

Collagenase type I, penicillin/streptomycin (Pen/Strep) broad-spectrum antibiotic cocktail, trypsin-EDTA (0.25%), fetal bovine serum (FBS), human insulin and Dulbecco’s Modified Eagle’s Medium (DMEM) were purchased from Gibco/Invitrogen (Carlsbad, CA, USA). VersaLyse^TM^ was purchased from Beckman Coulter (Miami, FL, USA). Dexamethasone, 3-isobutyl-methylxanthine, Nile Red (NR) and indomethacin were purchased from Sigma-Aldrich (St. Louis, MO, USA). Vybrant® DyeCycle^TM^ Violet was purchased from Thermo Fisher Scientific/Life Technologies (Eugene, OR, USA). The following mouse anti-human monoclonal antibodies were purchased from Biolegend (San Diego, CA, USA): CD14-APC Cy7 (Clone M5E2), CD31-PE Cy7 (Clone WM-59), CD36-APC (Clone 5-271), CD73-FITC (Clone AD2), CD44-APC Cy7 (Clone IM7) and CD105-PE (Clone 42A3). Mouse anti-human CD45-Krome Orange (Clone J.33), CD90-PE-Cy5 (Clone Thy-1), CD34-PE Cy7 (Clone 581), and the viability dye, 7-aminoactinomycin D (7-AAD) were purchased from Immunotech/Beckman Coulter (Marseille, France).

### Isolation of ASCs from adipose tissue

Adipose-derived stromal/stem cells (ASCs) were isolated from human adipose tissue as previously described^30,32^. Subcutaneous adipose tissue was obtained from healthy donors that underwent elective liposuction surgery under general anaesthesia. Informed consent was obtained from all donors. Samples were anonymized immediately after collection and only limited demographic information (age and gender) was supplied (Supplementary Table S1). The study was approved by the Research Ethics Committee, Faculty of Health Sciences, University of Pretoria (study numbers 218/2010, 421/2013 and 414/2015) and was conducted in accordance with the Declaration of Helsinki.

### Cryopreservation of ASCs at Passage (P) 0

Freezing medium was prepared by adding DMSO (10%) to complete DMEM. ASCs at P0 (P0 ASCs) refers to cells in the SVF with the ability to adhere to the surface of the tissue culture flasks after initial seeding. The adherent cells were allowed to reach 80–90% confluence after which they were dissociated as described below. After two washing steps (using PBS), cells were resuspended in freezing medium at a concentration of approximately 1 × 10^6^ cells/ml and transferred to cryovials (Greiner Bio-One, Kremsmünster, Austria). Cryovials were transferred to an isopropanol freezing container (Nalgene 1 °C/min freezing container, Thermo Fisher Scientific/ Nalge Nunc International, Penfield, NY, USA) for overnight freezing in a −80 °C freezer (Thermo Fisher Scientific, Waltham, MA, USA). After overnight freezing, the cryovials were transferred to a dewar (Thermo Fisher Scientific, Waltham, MA, USA) for storage in liquid nitrogen vapour. After 10–14 months in storage, the cryovials were removed from the dewar, and briefly thawed at room temperature to allow for quick transfer of the semi-frozen cell solution into 25 ml of pre-warmed (37 °C) complete DMEM culture medium. Cells were centrifuged at 300 g for 5 minutes. The cell pellet was washed once more with PBS and transferred to a T75 culture flask. The cells were maintained as described below. At P4, cells were seeded into 6-well plates (P5) and induced to differentiate into adipocytes as described below. The experimental design for assessing the effect of cryopreservation on the adipogenic differentiation of ASCs is summarized in Supplementary data Fig. S1.

### Expansion of ASCs

Cells were maintained at 37 °C/5% CO_2_ in complete DMEM culture medium. At 80–90% confluence, the cells were dissociated for 10 minutes at 37 °C using 0.25% trypsin-EDTA, and seeded at a density of 5 × 10^3^ cells/cm^2^ into T75 cultures flasks (for expansion) and 6-well plates (for adipocyte differentiation). Cultures were expanded for five passages. The experimental design for assessing the effect of passaging (*ex vivo* expansion) on the adipogenic differentiation of ASCs is summarized in Supplementary data Fig. S2.

### Immunophenotype and cell viability

Adipose-derived stromal cells (ASCs) were phenotyped at each passage. Prior to immunophenotype analysis, ASCs were stained with the following in Tube 1: CD34-PE Cy7, CD36-APC, CD45-Krome Orange, CD73-FITC and CD105-PE; and in Tube 2: CD44-APC Cy7, CD45-Krome Orange, CD73-FITC, CD90-PE Cy5 and CD105-PE. Cell viability was determined at each passage after staining with 7-AAD. Multi-parameter flow cytometry analyses were performed using a Gallios flow cytometer (Beckman Coulter, Miami, FL, USA). Single color staining tubes were used to correct for spectral overlap of the fluorochromes (color compensation). Flow cytometry data was analysed using Kaluza flow cytometry data analysis software (Version 1.5, Beckman Coulter, Miami, USA).

### *In vitro* adipocyte differentiation

*In vitro* adipogenic differentiation of ASCs was performed as previously described^30,32^.

### Adipocyte quantification using flow cytometry

Prior to staining, the cells were dissociated as described above. The cell suspension was carefully transferred (using a plastic Pasteur pipette) to 15 ml tubes. Due to the fragile nature of adipocytes, no washing steps were performed. One millilitre (1 ml) aliquots of the cell suspensions were stained with Nile Red (20 ng/ml final concentration), Vybrant® DyeCycle^TM^ Violet (VDC Violet; 2.5 µM final concentration) and mouse anti-human CD36-APC. After a 20 min incubation at room temperature, cells were analysed using a Gallios flow cytometer. Nile Red was excited by a 488 nm laser and fluorescent emission signals were collected using the FL2 (575/30 nm BP) detector. Non-induced ASCs were used to optimise the PMT voltage settings as well as to set the signal-to-noise threshold of the Nile Red fluorescence. After initial optimisation, all instrument settings were kept constant for the duration of the study. Flow Check^TM^ Pro (Beckman Coulter, Miami, USA) fluorospheres were run daily to validate instrument performance. Flow cytometry data was analysed using Kaluza flow cytometry data analysis software (Version 1.5, Beckman Coulter, Miami, USA).

### Fluorescence microscopy

Cells (both non-induced and induced) were cultured in 6-well plates as described above. Prior to fluorescence microscopy imaging, both non-induced and induced cultures were stained with Vybrant® DyeCycle^TM^ Violet (2.5 μg/ml final concentration) and NR (final concentration 50 ng/ml). After a 20-minute incubation period (37 °C/5% CO_2_), the culture medium was removed and the wells rinsed with PBS to remove non-adherent cells and unbound residual dye. Fluorescence images were captured using an AxioVert A1 inverted fluorescence microscope (Carl Zeiss, Gottingen, Germany) equipped with an AxioCam Cm1 camera (Carl Zeiss). Single channel images were captured and subsequently converted into overlay images. Nile Red was captured using Filter Set 9 (excitation BP 450–490, emission LP 515; Carl Zeiss) to visualise yellow-gold fluorescence and Vybrant® DyeCycle^TM^ Violet was captured using Filter Set 49 (excitation G 365, emission BP 445/50; Carl Zeiss) to visualise nuclei. Images were initially captured using AxioVision software (Version 4.8.2). In order to optimally visualise intracellular lipid droplets, all images were enhanced, but not manipulated, post-acquisition using Image J imaging software^33^. Enhancement of images was done by adjusting contrast and brightness settings.

### RNA Isolation and RT-qPCR

RNA was isolated and reverse-transcription-quantitative polymerase chain reactions (RT-qPCR) performed as previously described^30,32^. The primers (IDT, Coralville, IA, USA) and their sequences for the genes of interest as well as the reference genes used, are summarised in Supplementary Table S2.

### Statistical analysis

For RT-qPCR data, relative gene expression was calculated using the modified comparative CT method (ΔΔC_T_) as indicated below:

$$\Delta {C}_{T}={C}_{T}^{GOI(NI\,or\,IND)}-{C}_{T}^{REF}$$

where GOI indicates gene of interest (non-induced or induced sample) and REF indicates reference genes

The following reference genes were used: *PPIA, TBP, YWHAZ* (Table S1)

$$\Delta \Delta {C}_{T}=\Delta {C}_{T}^{IND}-\Delta {C}_{T}^{IN}$$

where IND refers to induced sample and NI refers to non-induced sample.

Relative fold-increases were calculated using amplification efficiencies determined for each of the genes of interest through standard curves.

$$Relative\,fold\,increase={E}^{-(\varDelta \varDelta Ct)}$$

where *E* refers to the amplification efficiency obtained for the specific gene of interest.

Results are expressed as mean ± standard deviation (SD) or standard error of the mean (SEM). Outliers were identified using the ROUT statistical test (Q = 0.1%) and excluded from statistical analysis. The Mann-Whitney test was used to determine statistical significance between groups. GraphPad PRISM 7 (Version 7.02, Graphpad Software, San Diego, USA) was used for all statistical analyses. Differences between groups were considered to be significant if the *P* values were ≤ 0.05, with *, **, and *** corresponding to *P* ≤ 0.05, *P* < 0.01, and *P* < 0.001, respectively.

## Results

In order for cells to be characterized as MSCs they need to be plastic adherent, express a pre-defined set of cell surface proteins and have the ability to differentiate into adipocytes, chondrocytes and osteoblasts^31^. Only adipogenic differentiation potential was investigated in this study.

ASCs were dislodged from cell culture vessel surfaces and cell viability assessed before being used in downstream experiments. No significant differences in viability were observed between non-induced and induced cultures or between different passages (Supplementary data Table S3). The percentage viability for all passages combined (P0 to P5) was 95.69 ± 2.85% and 96.08 ± 3.23%, for non-induced and induced samples, respectively.

**Figure 1:**
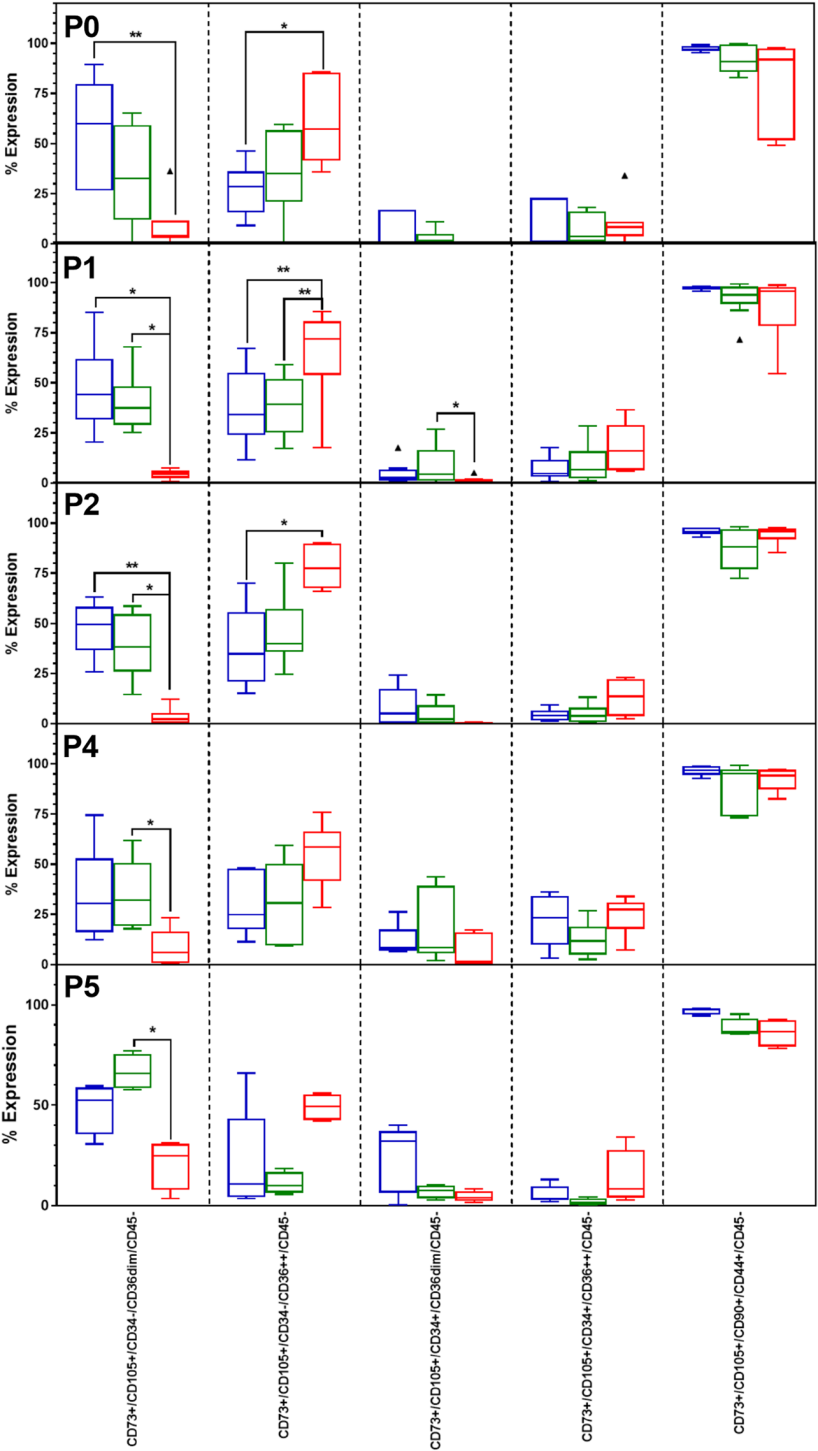
Phenotypic profiles of ASCs at various passages (P0, P1, P2, P4, P5) during *ex vivo* expansion. Results are displayed as Tukey box-whisker plots where the median value are indicated by the solid horizontal line in each box. Results are representative of 5 to 9 independent ASC cultures. Blue boxes indicate the percentage expression observed for non-induced cells, green boxes represent induced ASCs which did not differentiate into adipocytes, and red boxes represent induced ASCs which differentiated into adipocytes (increased SS). Significance between non-induced and induced (not differentiated and differentiated) cultures is indicated by an asterisk. *P < 0.5; **P < 0.01. ▲ indicates outliers as identified by the Tukey box-whisker plot rules. The P3 data set contained less than 5 data points and was excluded.

All undifferentiated ASCs used in this study were positive for CD44, CD73, CD90 and CD105 and negative for CD45. No significant differences were observed between the percentage of ASCs displaying the above-mentioned phenotype at the various passages (Fig. 1; last column). The proportion of ASCs displaying a CD44^+^/CD73^+^/CD90^+^/CD105^+^/CD45^−^ co-expression profile was 97.28 ± 1.34% at P0, 97.23 ± 0.83% at P1, 95.78 ± 1.93% at P2, 96.53 ± 2.31% at P4 and 96.95 ± 1.74% at P5 (Fig. 1; last column). In contrast to the uniformity observed for the markers mentioned above, CD34 and CD36 expression varied at the various passages. The respective sub-populations present at the various passages, however, did not differ significantly between passages. As we have previously reported^30^, the majority of ASCs express CD36 dimly (CD36^dim^) with a sub-population of cells that express CD36 at a higher intensity (CD36^++^) (Fig. 1). The main phenotypic difference between undifferentiated and differentiated (adipogenic) ASCs was the upregulation of CD36 expression during adipocyte differentiation (Fig. 1), confirming the findings reported by several other groups that CD36 is associated with adipocyte differentiation^30,34–36^.

**Figure 2:**
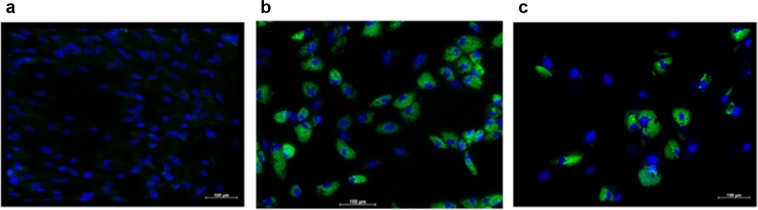
Representative fluorescence microscopy images of undifferentiated (**a**) and differentiated ASCs (**b**,**c**). Cells were stained with Nile Red (green; lipid droplets) and VDC Violet (blue; nuclei). (**a**) ASCs that were not induced to differentiate into adipocytes; (**b**) Differentiated ASCs at P0; (**c**) Differentiated ASCs at P5. ASCs were not cryopreserved prior to adipogenic differentiation. All three images are from the same primary ASC culture. Images were captured 21 days post induction as two single channel images which were then merged using Image J software. 10 x magnification.

Intracellular lipid droplet accumulation is a key morphological feature associated with adipocyte differentiation^26,37^ (Fig. 2a,c). Intracellular lipid droplet accumulation increases the side scatter (SS) signal (indicative of cellular complexity) of the cells when analysed by flow cytometry (Supplementary data Fig. S3). Not all ASCs exposed to adipogenic induction medium differentiated into adipocytes (Figs 1 and 2). Interestingly, ASCs exposed to adipogenic differentiation medium but which did not proceed to full adipogenic differentiation (no detectable intracellular lipid droplet accumulation based on their SS profiles) displayed similar phenotypic profiles as non-induced cells (Fig. 1).

*Ex vivo* expansion and cryopreservation are often used in the laboratory either to increase cell numbers or preserve cells for future use. Ideally, ASCs should maintain their therapeutic potential, including differentiation potential, during *ex vivo* expansion and cryopreservation. In the first series of experiments we investigated the effect of cryopreservation on the expansion rate and the ability of ASCs to undergo adipogenic differentiation. ASCs (P0) were cryopreserved for 10–14 months after which the cells were removed from cryostorage and expanded to P5 (Supplementary Fig. S1). At P5, ASCs were induced to differentiate into adipocytes. The level of adipocyte differentiation observed in the previously cryopreserved ASCs was compared to the degree of adipocyte differentiation observed for ASCs (P5) from the same culture that had not previously been cryopreserved.

The relative expansion rate was calculated as the expansion period (days) required for ASC cultures to reach 80–90% confluence at each passage. As confluence estimates can be subjective, and in an attempt to minimize potential error in judgment, confluence estimates were performed by the same person for the duration of the study.

The formula used was:

1 $$Relative\,expansion\,period\,(days)=\tfrac{(Number\,of\,days\,in\,culture,i.e.\,at\,P1)\ast \,\log (2)}{\log (total\,number\,of\,viable\,cells\,harvested,\,i.e\,P1)-\,\log (total\,number\,of\,viable\,cells\,seeded,i.e\,P1)}$$

**Figure 3:**
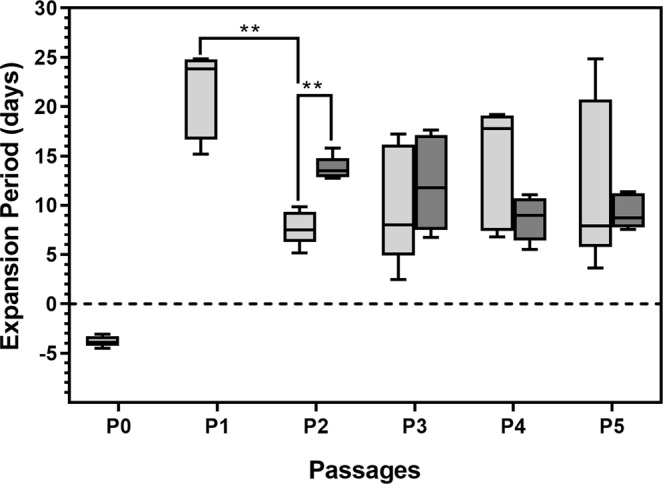
Relative expansion periods (days) required to achieve 80–90% confluence at various passages (P0–P5). Results are displayed as minimum/maximum box-whisker plots where the median is indicated by the solid horizontal line in each box. Results represent 5 independent ASC cultures. Results from ASC cultures that did not undergo cryopreservation are indicated by light grey boxes, while dark grey boxes represent results from the same ASC cultures that underwent cryopreservation at P0. **P < 0.01.

The average number of days from initial seeding (P0) to replating (P1) was 7.4 ± 0.9 days. The total number of cells harvested at P0 confluence (80–90%) was lower than the initial seeding density (5 × 10^5^ nucleated SVF cells/cm^2^), resulting in an initial period of negative expansion when the equation was applied (Fig. 3). At P1, the relative expansion period required for the primary ASC cultures to reach 80–90% confluence was 21.34 ± 4.41 days, which was significantly (p = 0.013, n = 5) longer than the relative expansion period (7.73 ± 1.77 days) required to achieve confluence at P2 (Fig. 3). No significant differences were observed between expansion periods observed at P2, P3 (10.01 ± 6.04 days), P4 (14.16 ± 6.20 days) and P5 (12.17 ± 8.51 days) (Fig. 3).

The relative expansion periods required for previously cryopreserved ASCs to reach 80–90% confluence were monitored at P2, P3, P4 and P5. A significantly (p = 0.008, n = 5) longer expansion period (13.76 ± 1.22 days) was observed for ASCs that underwent cryopreservation at P2 when compared to the relative expansion period (7.73 ± 1.77 days) observed at P2 for ASC cultures not previously cryopreserved (Fig. 3). However, no significant differences in the relative expansion period were observed at P3, P4 and P5 between cultures that did and did not undergo cryopreservation (Fig. 3). The relative expansion periods observed for previously cryopreserved ASC cultures were 12.21 ± 4.87 days at P3, 8.65 ± 2.27 days at P4 and 9.34 ± 1.77 days at P5 (Fig. 3).

**Figure 4:**
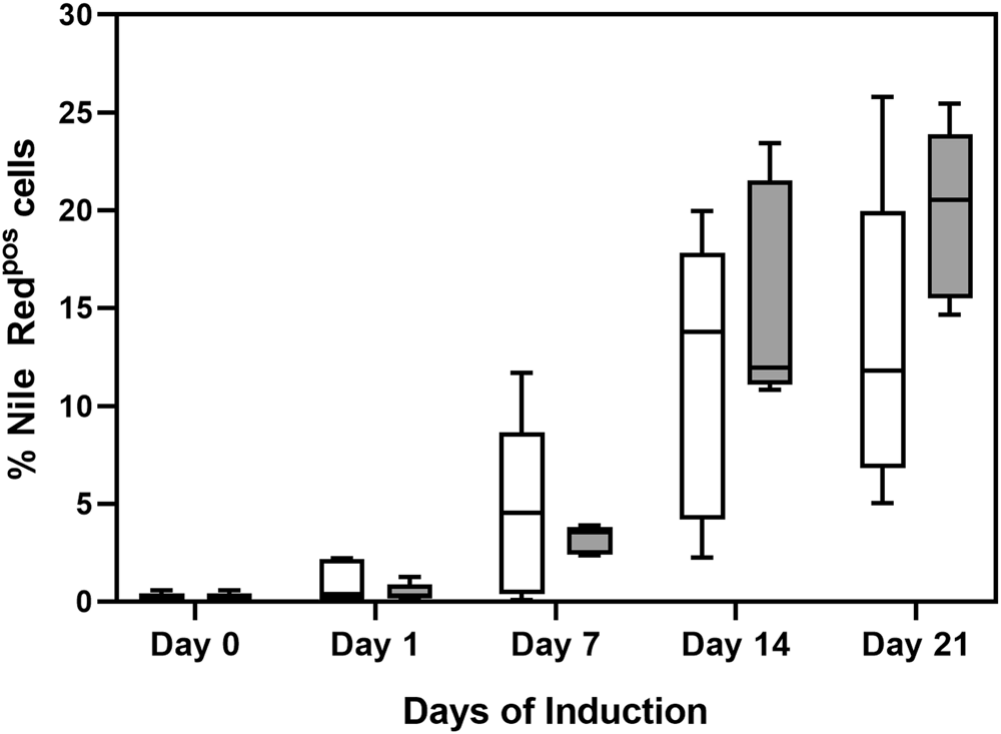
Proportion of ASCs (P5) with increased levels of intracellular neutral lipid content (Nile Red-positive). White boxes represent results of ASCs not previously cryopreserved, while grey boxes represents results of ASCs previously cryopreserved at P0. Cultures were terminated on days 0, 1, 7, 14 and 21 during a 21-day adipogenic induction period. Data represents 5 independent ASC cultures. Results are displayed as Tukey box-whisker plots where the median is indicated by the horizontal line in each box.

Adipogenic differentiation was assessed by investigating gene expression levels of key adipogenesis-associated genes as well as quantifying the proportion of differentiated ASCs with increased CD36 expression and intracellular lipid content at various time points during the 21-day differentiation period. No significant differences were observed in adipocyte differentiation at P5 between cells that did and did not undergo cryopreservation (Fig. 4). ASCs with detectable levels of intracellular lipid accumulation were observed from day 7 onwards for both non-cryopreserved and cryopreserved ASCs (Fig. 4). At day 7, 4.53 ± 4.65% and 3.20 ± 0.74% of induced non-cryopreserved and cryopreserved ASCs displayed increased levels of intracellular neutral lipid accumulation. The percentage of induced ASCs (not exposed and exposed to cryopreservation) increased on day 14 to 11.57 ± 7.22% and 15.43 ± 5.74%, respectively (Fig. 4). On day 21, the percentage of cells with increased intracellular neutral lipid accumulation increased to 13.07 ± 7.88% for non-cryopreserved ASCs and to 19.85 ± 4.40% for cryopreserved ASCs (Fig. 4). Less than 1% of the non-induced (control) ASCs displayed detectable levels of intracellular neutral lipid accumulation at day 0. No noticeable intracellular neutral lipid accumulation was observed in non-induced (control) ASCs throughout the 21-day culture period (Fig. 2a).

**Figure 5:**
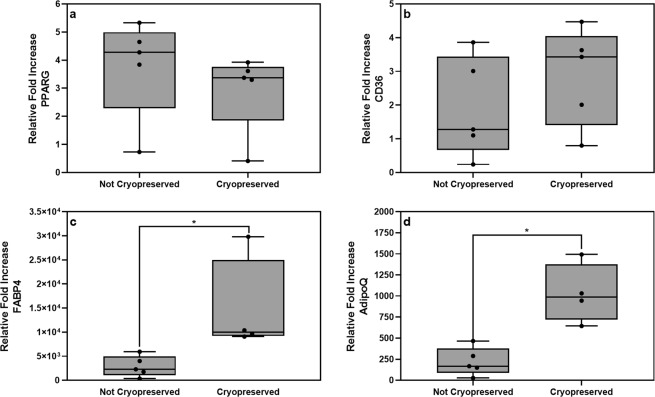
Relative fold increase in mRNA expression levels of adipogenesis-associated genes as determined by RT-qPCR at day 21 post adipogenic induction. (**a**) *PPARG*, (**b**) *CD36*, (**c**) *FABP4* and (**d**) AdipoQ. Gene expression levels were normalized to the following reference genes: *PPIA, TBP, YWHAZ* (ΔCt), after which they were normalized to control (non-induced) cells (ΔΔCt). Results are displayed as minimum/maximum box-whisker plots in which the median is indicated by a solid horizontal line in each box. Results are representative of 5 independent ASC cultures. Significance in relative gene expression levels between non-cryopreserved and cryopreserved cultures is indicated with asterisks. *p < 0.05.

The transcription factor, peroxisome proliferator-activated receptor gamma (*PPARG*), was upregulated in induced cells when compared to the relative gene expression levels detected in non-induced cells (data not shown). There were however no noticeable differences in *PPARG* gene expression levels between non-cryopreserved and cryopreserved cultures when gene expression was normalized to the corresponding non-induced cells (Fig. 5a). Upregulation of *PPARG* is essential for the subsequent upregulation of end-stage adipogenic genes, such as *CD36*, *FABP4* and *ADIPOQ*. Interestingly, higher gene expression levels were observed for these end-stage genes in cultures previously cryopreserved (Fig. 5b–d). Relative *FABP4* (p = 0.016) and *ADIPOQ* (p = 0.016) gene expression levels (normalised to corresponding non-induced cultures) were significantly higher in cryopreserved ASCs when compared to cultures not previously cryopreserved (Fig. 5c,d).

**Figure 6:**
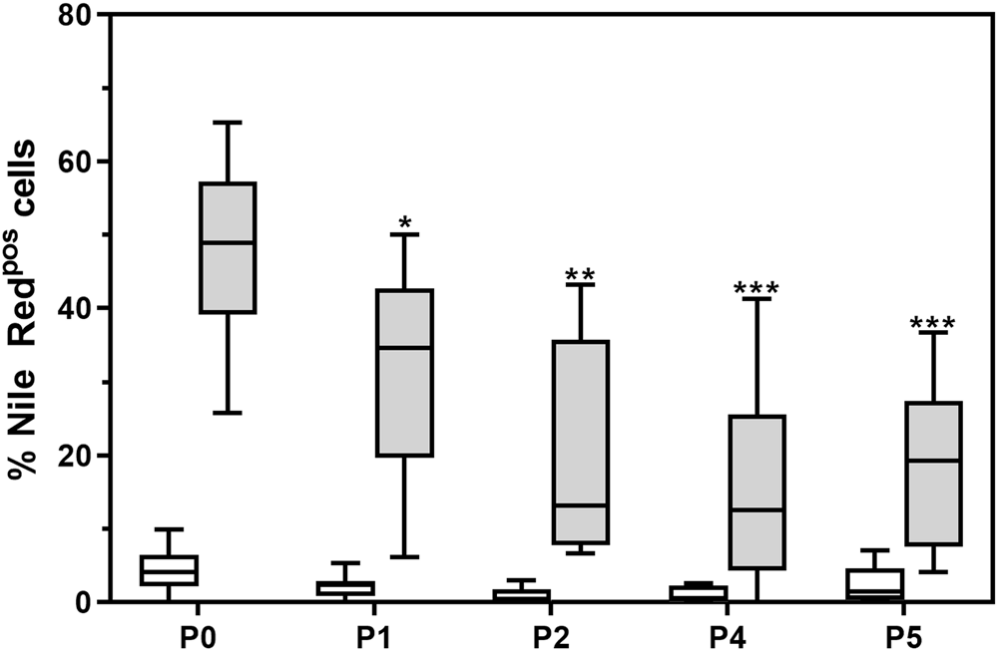
Proportion of intact cells with increased levels of intracellular neutral lipid content (Nile Red-positive) during initial rounds (P0 to P5) of *ex vivo* expansion. Cultures were terminated 21 days after induction of adipocyte differentiation. Data represent primary ASC cultures from 10 different donors. White and grey boxes indicate results from non-induced and differentiated cells (adipocytes). Significance when compared to P0 is indicated by asterisks. *P < 0.5; **P < 0.01; ***P < 0.001. Results are displayed as Tukey box-whisker plots where the median is indicated by the horizontal line in each box. The P3 data set contained less than 5 data points and was excluded.

We also investigated the impact of *ex vivo* expansion on the ability of ACS to undergo adipogenic differentiation. ASCs used in this set of experiments had not previously been cryopreserved. At P0, 47.29 ± 12.18% of differentiated ASCs displayed an increase in intracellular neutral lipid content (Fig. 6). The proportion of ASCs that were able to differentiate into adipocytes decreased to 36.79 ± 5.75% at P1, 24.02 ± 15.94% (p = 0.0377, compared to P0) at P2, 19.64 ± 14.32% (p = 0.0068 compared to P0) at P4 and 21.95% ± 10.68 (p = 0.0092 compared to P0) at P5 (Fig. 6). The percentage of non-induced cells with detectable levels of intracellular neutral lipid was less than <5% (Fig. 6), and no significant changes in lipid accumulation were observed in these cells over the 21-day culture period.

**Figure 7:**
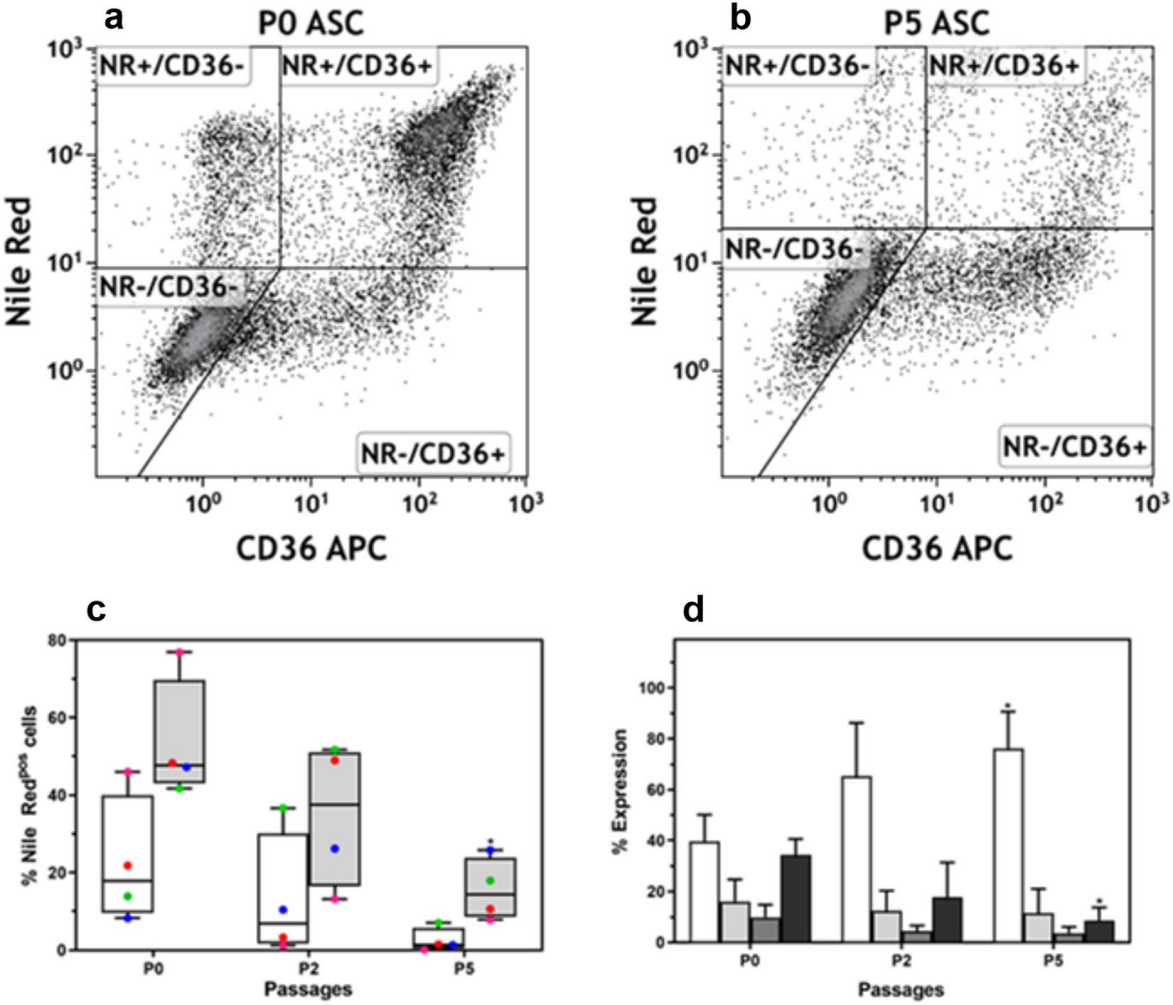
The impact of *ex vivo* expansion on adipogenic differentiation-associated CD36 expression. (**a**,**b**) Representative flow cytometry density plots to illustrate the distribution of four adipocyte sub populations based on the level of intracellular lipid accumulation (NR^+^/CD36^−^ and NR^+^/CD36^+^) combined with the absence or presence of CD36 cell surface protein expression (NR^−^/CD36^+^ and NR^+^/CD36^+^). (**a**) A representative flow cytometry density plot indicating the four sub-populations observed at P0. (**b**) A representative flow cytometry density plot indicating the four sub-populations observed at P5. (**c**) Percentage ASCs expressing intermediate levels of CD36 (CD36^++^). Results are expressed as minimum/maximum box-whisker plots. The median is indicated by a solid horizontal line within each box. White and grey bars represent non-induced and induced cells (adipocytes), respectively. Cultures were terminated 21 days after induction of adipocyte differentiation. *P < 0.5 and indicates significance between % expression observed at P0 and P5. Four independent ASC cultures were studied, indicated by different coloured symbols. (**d**) Impact of early rounds of *ex vivo* expansion on the distribution of adipocyte sub-populations present at day 21 of *ex vivo* adipocyte differentiation. Mean percentage expression ± standard deviation (SD) of the four adipocyte sub-populations as observed at P0, P2 and P5. White bars represents the Nile Red (NR)^−^/CD36^−^ sub-population; light grey bars represent the NR^+^/CD36^+^ sub-population; dark grey bars represent the NR^−^/CD36^+^ sub-population; and black bars represent NR^+^/CD36^−^ sub-population. *P < 0.5 and indicates significance between % expression observed at P0 and P5.

Adipocyte differentiation is associated with upregulation of CD36 cell surface expression which precedes intracellular lipid accumulation (Fig. 7a,b)^30^. *In vitro* data suggest that intracellular lipid accumulation only occurs when the maximum level (based on fluorescence intensity; maximum right shift) of CD36 cell surface expression is reached (Fig. 7a,b)^30^. We observed that the total proportion of ASCs (undifferentiated and differentiated) expressing intermediate/high levels of CD36 (CD36^++/+++^) gradually decreased as the number of *ex vivo* expansion rounds increased (Fig. 7a,b), reaching significance (p = 0.029, n = 4; when compared to P0), for both undifferentiated and differentiated cells at P5 (Fig. 7c). At P0, 22.50 ± 16.63% of undifferentiated ASCs expressed intermediate/high levels of CD36 (Fig. 7c). The percentage of undifferentiated ASCs expressing intermediate/high levels of CD36 decreased to 12.92 ± 16.32% at P2 and 2.50 ± 3.10% at P5 (Fig. 7c). The proportion of differentiated ASCs that expressed intermediate/high levels of CD36 was significantly higher, when compared to CD36^++/+++^ expression observed for undifferentiated cells. At P0, 53.53 ± 15.83% (p = 0.057, n = 4 when compared to undifferentiated cells) of differentiated ASCs expressed intermediate/high levels of CD36. The percentage of differentiated ASCs expressing intermediate/high levels of CD36 decreased to 35.01 ± 18.51% at P2 and 15.58 ± 8.06% at P5 (p = 0.029, when compared to undifferentiated cells) (Fig. 7c).

Combining Nile Red staining (intracellular lipid detection) and CD36 cell surface staining allows the identification of three distinct populations during adipocyte differentiation, namely undifferentiated ASCs (Nile Red^Neg^/CD36^Neg^), ASCs with increased CD36 cell surface expression, but no detectable levels in intracellular lipid accumulation (Nile Red^Neg^/CD36^Pos^) and differentiated ASCs (Nile Red^Pos^/CD36^Pos^) (Fig. 7a,b). Interestingly, we observed a fourth population in this study (Nile Red^Pos^/CD36^Neg^) that was present at a higher frequency in early passages (P0) compared to later passages (P5) (Fig. 7a,b). We observed that all four sub-populations gradually decreased as the cells were expanded *ex vivo* (Fig. 7D). The Nile Red^Pos^/CD36^Pos^ sub-population reached significance (p = 0.057, n = 4) at P5 (Fig. 7D). In contrast, we observed a gradual increase in the frequency of the undifferentiated (Nile Red^Neg^/CD36^Neg^) cell population during *ex vivo* expansion reaching significance (p = 0.029, n = 4) at P5 (Fig. 7d).

**Figure 8:**
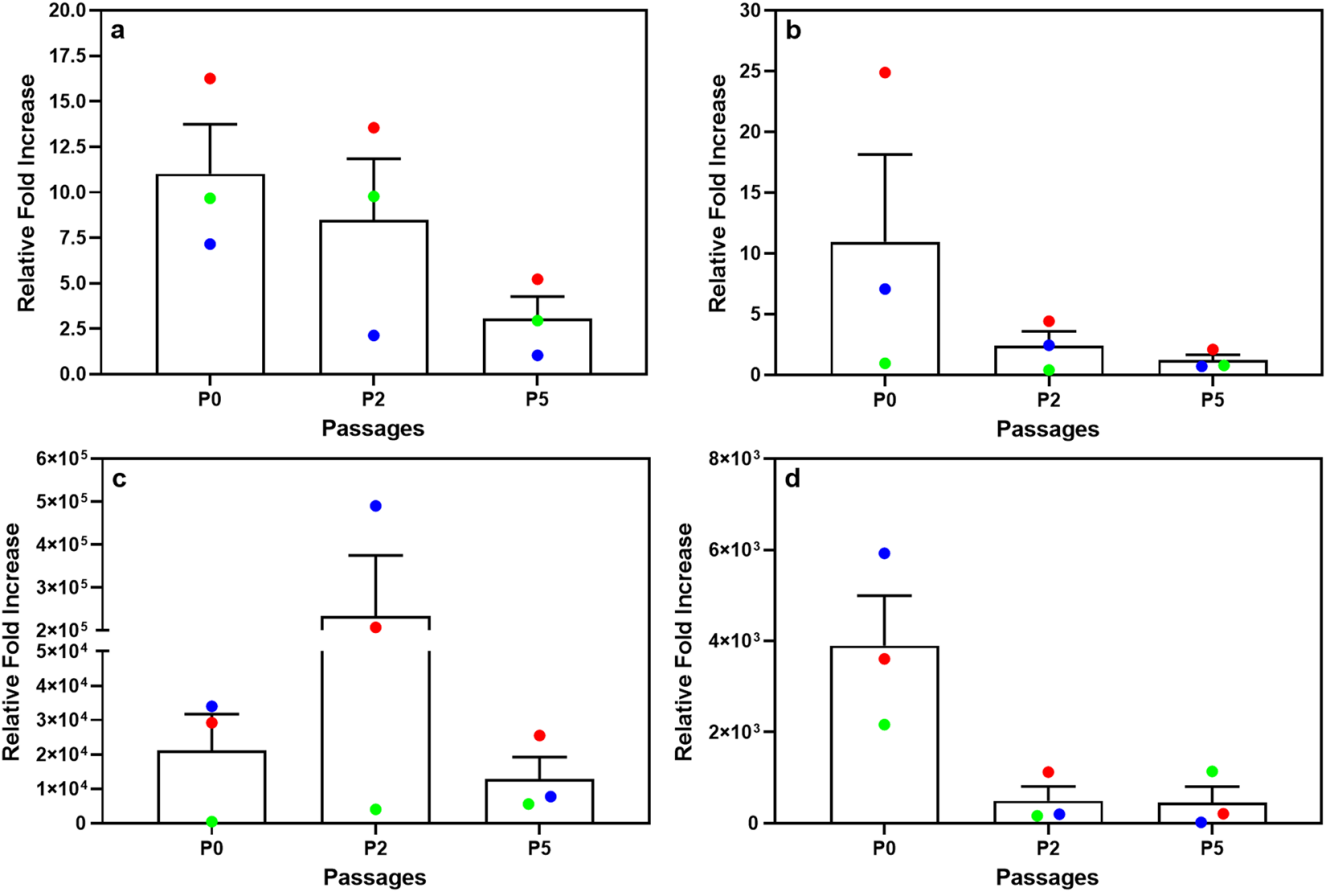
Relative fold increase in mRNA expression levels of adipogenesis-associated genes at P0, P2 and P5. Results are expressed as relative fold-change in gene expression when normalized to the respective undifferentiated control cells. (**a**) *PPARG*; (**b**) *CD36*; (**c**) *FABP4* and (**d**) *ADIPOQ*. All induced samples were first normalized to reference genes (*PPIA, TBP, YWHAX*) (ΔC_T_) followed by normalization against the respective undifferentiated (non-induced) samples (ΔΔC_T_). Three independent ASC cultures were studied, indicated by different coloured symbols.

The relative levels of expression of adipogenesis-associated genes were also investigated (Fig. 8). A gradual decrease in *PPARG*, *CD36* and *ADIPOQ* gene expression (relative fold increase, normalized to corresponding non-induced cultures) was observed with increasing rounds of *ex vivo* expansion (Fig. 8a,b,d). However, none of the decreases observed were significant. The lack of significance is likely due to the high degree of inter-culture variability observed. Interestingly, we observed an increase in FABP4 gene expression at P2 for two of the cultures studied (Fig. 8c). The reason for the upregulation of FABP4 at P2 is unclear.

## Discussion

Adipose tissue, often harvested in relatively large quantities during cosmetic surgical procedures such as lipoaspirate and abdominoplasty, is a rich source of multipotent ASCs^38^. Adipose tissue harvested during these procedures is considered biological waste and is thus associated with minimal ethical concerns. All of these factors make adipose tissue an increasingly popular source from which multipotent MSCs can be harvested for therapeutic use.

Various studies have shown that donor age, body mass index (BMI) and the general state of health of the donor might have an impact on the therapeutic potential of ASCs^39^. Collectively, these studies suggest that ASCs isolated from young, healthy, lean donors hold the greatest potential for clinical benefit. It would thus be beneficial if ASCs with enhanced therapeutic potential could be isolated, expanded and cryopreserved for future clinical applications. However, it is important to understand the impact of both cryopreservation and *ex vivo* expansion on the differentiation potential of these cells^40^. DiGirolamo and colleagues (1999) showed that osteogenic differentiation was maintained during *ex vivo* expansion of bone marrow-derived MSCs, while the potential of these cells to undergo adipogenic differentiation decreased during expansion^41^. Halleux and colleagues (2001) also showed that clones of bone marrow-derived MSCs fail to undergo adipogenic and chondrogenic differentiation after *ex vivo* expansion^42^. Using bovine adipose-derived stromal cells, Zhao and colleagues (2012) showed that *ex vivo* expansion resulted in a decrease in adipogenic and chondrogenic differentiation potential, but not osteogenic differentiation potential^43^. Safwani and colleagues (2011) showed, using human ASCs, that extensive *ex vivo* expansion (≥P5) resulted in decrease expression of adipogenic differentiation associated genes^44,45^.

The ability of ASCs to differentiate into adipocytes not only allows for the study of the process of adipogenesis *in vitro*, but may also contribute to the clinical application of ASC-associated cell therapy products. One such clinical application is fat grafting, a well-established technique often used in soft tissue reconstruction/augmentation procedures^11^. In clinical practice, fat grafts are often supplemented with stem cells, more specifically ASCs, a practice commonly referred to as cell-assisted lipotransfer (CAL)^46,47^. The enhanced benefits observed using CAL are contributed to by the ability of ASCs to differentiate into adipocytes, resulting in enhanced volume retention of the fat graft^46,47^.

In this study, adipocytes were characterized based on their expression of CD36 cell surface protein and increased intracellular lipid levels. We showed in a recent study^30^, and confirmed in this study, that CD36 expression is upregulated during adipocyte differentiation and precedes intracellular lipid accumulation. We also observed that increasing rounds of *ex vivo* expansion results in a gradual decline in the proportion of ASCs that display increased CD36 expression levels and a consequent decrease in intracellular lipid accumulation during adipogenic differentiation, i.e. in ASCs that differentiated into adipocytes. CD36, a fatty acid translocase, plays an important role during adipocyte differentiation and upregulation of CD36 expression^30,35,48^. Gao *et al*. (2017) suggested that isolation of CD36-expressing cells from SVF, would select for cells with enhanced adipogenic potential^35^.

All ASCs used in this study uniformly expressed CD73, CD44, CD90 and CD105, while not expressing CD45, a cell surface protein associated with mature leukocytes. The ASCs used in this study therefore adhered to the phenotypic cell surface expression profile, with the exception of CD34, recommended by the International Federation for Adipose Therapeutics and Science (IFATS) and the International Society for Cellular Therapy (ISCT)^31^. In contrast to the uniformity of expression observed for these markers and aligned with several recent reports^15,20,49–51^, we observed variable expression of the phosphoglycoprotein, CD34. The function of CD34 in MSCs is largely unknown. Suga and colleagues (2009) isolated CD34-negative and CD34-positive ASCs sub-populations and investigated their respective biological functions^52^. They observed differences in the proliferation rate, differentiation potential and gene expression profiles between the two sub-populations and concluded that the CD34-positive ASC population is more immature (undifferentiated) and that the loss of CD34 expression is a function of ASC commitment to undergo differentiation^52,53^. Other investigators oppose this view and suggest that the variable expression of CD34 observed *ex vivo* is due to the cells being cultured in sub-optimal *ex vivo* conditions, which lack the factors necessary to support CD34 expression *in vivo*^50^.

The reason for the *ex vivo* expansion-associated decrease in ASC adipogenic potential is unknown. Selective loss of ASC sub-populations with adipogenic differentiation potential during culturing is a possible explanation. Selich and colleagues (2016) showed, using umbilical cord-derived MSCs, that a significant level of clonal selection occurs during the *in vitro* expansion of these cells^54^. Post and colleagues (2008), using MSCs isolated from murine bone marrow, suggested that bone marrow-derived MSCs contain precursors that are committed either to adipogenic or osteogenic differentiation^55,56^. It is possible that similar progenitors are present during the early (i.e. P0 and P1) passages of ASC cultures, and that these are gradually lost during extended *ex vivo* expansion, resulting in the diminished adipocyte differentiation potential of *ex vivo* expanded ASCs. Another explanation may be the gradual loss of cells, such as adipose tissue-associated macrophages (ATMs), which are known to play an important role in adipose tissue homeostasis. Adipose tissue-associated macrophages (ATMs) are one of the more abundant cell types present in adipose tissue^57,58^. As a result of their ability to adhere to plastic, ATMs are present as a relatively large proportion of the total cell population during the initial passages of ASC culture^57^ (Supplementary Fig. S4). Several *in vitro* studies suggest that there is crosstalk between ATMs and ASCs^59–61^ and that ATMs may influence the adipogenic differentiation potential of ASCs^58,62–64^. The majority of these studies suggest that ATMs suppress adipogenic differentiation *in vitro*, but the exact impact of ATMs on ASC adipogenic differentiation is not fully understood. In a recent study, He and colleagues (2018) showed that cell culture supernatant from M1 (pro-inflammatory) macrophage cultures promoted adipogenic differentiation, while culture supernatant from M0 (undifferentiated) and M2 (anti-inflammatory) macrophages suppressed adipogeneic differentiation^65^. Contrary to these findings, Lee and colleagues (2016) suggested that CD44 positive-M2 macrophages promote adipogenic differentiation^62^. These authors suggest that the catalytic products, 9-hydroxyoctadecadienoic acid (9-HODE) and 13-hydroxyoctadecadienoic acid (13-HODE) of arachidonate 15-lipoxygenase (Alox15), an enzyme highly expressed by M2 macrophages, promote CD36-mediated uptake of oxidised low-density lipoprotein (OXLDL), and in so doing contribute to intracellular lipid accumulation^62^. These authors further suggest that 9-HODE and 13-HODE drive the differentiation of white adipose precursors into brown adipocytes^62^.

We also investigated the impact of cryopreservation on the adipocyte differentiation potential of ASCs that underwent a limited number of rounds of *ex vivo* expansion. We did not observe a significant change in the ability of ASCs to undergo adipocyte differentiation in cells that had been cryopreserved at P0 for 10–14 months. Our observation is in line with similar observations made by several other investigators^40,66–68^. Lee and co-workers^66^ cryo-stored unprocessed lipo-aspirates for up to one year and found no significant difference in the adipogenic differentiation potential of ASCs isolated from these lipo-aspirates and expanded to P1/P2, when compared to the adipogenic differentiation potential of ASCs isolated from freshly harvested lipo-aspirate tissue. Goh and co-workers^68^ cryopreserved ASCs at P1 for at least one month, after which the cells were removed from cryo-storage, followed by immediate assessment of their adipogenic differentiation potential. These investigators found that cryopreservation had no significant effect on the proliferation rate or the adipocyte differentiation capabilities of ASCs. Gonda and co-workers^40^ and Kokai and co-workers^67^ likewise found no decrease in the adipocyte differentiation potential of ASCs after cryopreservation (for 6 months and 10 years, respectively). Interestingly, we observed higher relative levels of *FABP4* and *AdipoQ* gene expression in ASCs that were cryopreserved at P0. To our knowledge, only a limited number of studies have investigated the effect of cryopreservation on the expression of adipogenic-associated genes using ASC cultures from the same donor. Yong and colleagues^69^ investigated the effect of cryopreservation (3 months) on the phenotypic expression, proliferation rate and differentiation potential of ASCs. They found that cryopreservation had no impact on the proliferation rate and differentiation potential, including adipogenenic differentiation potential^69^. In our study the increased expression of end-stage adipogenic genes, such as *FABP4* and *AdipoQ*, observed in cryopreserved ASC cultures correlates with the slightly higher level of adipogenic differentiation observed in these cultures at day 21 post-induction. The reasons for the higher levels of *FABP4* and *AdipoQ* expression observed in ASC cultures previously cryopreserved requires further investigation. Duan and Lopez (2016), using canine multipotent stromal cells, suggested that cryopreservation changes the expression profiles of these cells^70^. Shaik and colleagues (2017) showed that the induction of heat shock proteins (HSPs) enhances ASC stemness^71^. It has been reported that cold shock increases the expression of HSPs^72^. It is therefore possible that HSPs are upregulated during the initial phases of cryopreservation, which in turn enhances the multipotent characteristics of ASCs when they are thawed and expanded post-cryopreservation. However, this hypothesis needs experimental confirmation.

Our data suggest that cryopreservation does not have a significant impact on the rate of ASC expansion. As expected, cryopreserved cells initially took slightly longer to reach 80–90% confluence. This may in part be due to the exposure of these cells to DMSO. DMSO was diluted and removed as soon as the cells were removed from liquid nitrogen storage. However, it may still affect the proliferation ability of these cells. The cells, however, are soon restored to normal function and no significant differences in the periods required to expand these cells to P5 were observed when non-cryopreserved ASCs (54.2 ± 6.4 days, P2 to P5) were compared to those that had been cryopreserved (58.2 ± 1.1 days, P2 to P5).

In summary, we have investigated the effect of ex *vivo* expansion (P0–P5) on the adipocyte differentiation potential of ASCs as well as which pre-adipocyte sub-populations are affected during the process. We also investigated if cryopreservation soon after isolation (P0) affects the adipocyte differentiation potential of ASCs when expanded *ex vivo*. Our data suggest that cryopreservation does not affect the ability of ASCs to differentiate into adipocytes. However, we found that an ASC sub-population that upregulates CD36 expression when induced to differentiate into adipocytes, decreased with increasing rounds of *ex vivo* expansion, resulting in a gradual decrease in the ability of ASC cultures to undergo adipogenic differentiation.

## Supplementary information

Supplementary Data

## Data Availability

The data is available on request.
