# Supplementary material for: The Effect of Early Rounds of ex vivo Expansion and Cryopreservation on the Adipogenic Differentiation Capacity of Adipose-Derived Stromal/Stem Cells
Source: Sci Rep. 2019 Nov 4;9:15943. doi: 10.1038/s41598-019-52086-9 (PMC6828715; doi:10.1038/s41598-019-52086-9)
Supplement: Supplementary file 1 — Supplementary Data [file 41598_2019_52086_MOESM1_ESM.pdf]

SUPPLEMENTARY DATA

THE EFFECT OF EARLY ROUNDS OF *EX VIVO* EXPANSION AND CRYOPRESERVATION ON THE ADIPOGENIC DIFFERENTIATION CAPACITY OF ADIPOSE-DERIVED STROMAL/STEM CELLS

Durandt, C.[*]; Dessels, C.; da Silva, C; Murdoch, C. Pepper, M.S.

Institute for Cellular and Molecular Medicine, Department of Immunology, and SAMRC Extramural Unit for Stem Cell Research and Therapy, Faculty of Health Sciences, University of Pretoria, Pretoria, South Africa.

Supplementary Table S1: Lipo-aspirate donor information

Date Obtained	Sample ID	Source	Donor Age at collection (Years)	Gender
2014-06-26	A260614	Abdominal	33	Female
2015-02-10	A100215	Gluteal	NA	Female
2015-04-07	A070415	Abdominal	44	Female
2015-05-26	A260515	Abdominal	NA	Female
2015-09-01	A010915	Gluteal	40	Female
2015-12-08	A081215_01	Abdominal	NA	Female
2016-01-12	A120116_01	Gluteal & Inner thigh	NA	Female
2016-01-26	A260116_01	Abdominal	21	Female
2016-01-26	A260116-02	Abdominal	36	Male
2017-04-27	A270417	Abdominal	NA	Female
2017-06-20	A200617	Abdominal	NA	Female
2017-06-28	A280617	Abdominal	NA	Female
2017-07-10	A100717	Abdominal	NA	Female
2017-08-15	A150817-01A	Abdominal	35	Female

NA: Not supplied

Supplementary Table S2: Primer Pairs

Gene		Gene Function	Accession Number	Primer pair sequences (5’ to 3’)	Amplicon Length (bp)	Tm (°C)	Efficiency (E)
Genes of Interest (GOI)							
Peroxisome proliferator-activated receptor gamma	*PPARG*	Regulates adipocyte transcription	NM_138712.3	F: CGTGGATCTCTCCGTAAT<br>R: TGGATCTGTTCTTGTGAATG	124	58	1.958
CD36/fatty acid translocase (FAT)	*CD36*	Facilitates long-chain fatty acids uptake	NM 001001548.1	F: CTTTGCCTCTCCAGTTGAA<br>R: ACACAGGTCTCCCTTCTT	122	58	1.967
Fatty acid binding protein 4	*FABP4*	Fatty acid uptake, transport, and metabolism	NM_001442.2	F: ATCAACCACCATAAAGAGAAA<br>R: AACTTCAGTCCAGGTCAA	126	58	1.953
Adiponectin	*ADIPOQ*	Encodes proteins involved with metabolic and hormonal processes	NM_001177800.1	F: GCCTGTTTCTGACCAATC<br>R: CCACTCTCCTATTTCTGATAAC	135	58	1.960
Reference Genes							
Peptidylprolyl isomerase A	*PPIA*	Protein folding through isomerization of oligopeptides	NM_001300981.1	F: GAGTTAAGAGTGTTGATGTAGG<br>R: CCTGGGACTGGAAAGTAA	116	58	2.600
TATA binding protein	*TBP*	RNA polymerase II transcription factor	NM_001172085.1	F: CCGAAACGCCGAATATAA<br>R: GGACTGTTCTTCACTCTTG	130	58	2.211
Tyrosine 3-Monooxygenase/Tryptophan 5-Monooxygenase Activation Protein, Zeta	*YWHAZ*	Signal transduction	NM_001135699.1	F: TGACATTGGGTAGCATTAAC<br>R: GCACCTGACAAATAGAAAGA	126	58	1.976

Cell Viability

Supplementary Table S3: Percentage viability of non-induced and induced ASC cultures at the various passages

PASSAGES	% VIABILITY			
	NON-INDUCED		INDUCED	
	Mean ± SD	n	Mean ± SD	n
**P0**	96.39 ± 1.32	11	96.33 ± 1.50	11
**P1**	96.76 ± 2.09	12	97.22 ± 1.46	12
**P2**	97.73 ± 1.07	10	94.78 ± 6.52	10
**P4**	96.15 ± 2.04	5	95.29 ± 1.66	5
**P5**	93.94 ± 3.08	10	95.93 ± 2.23	10

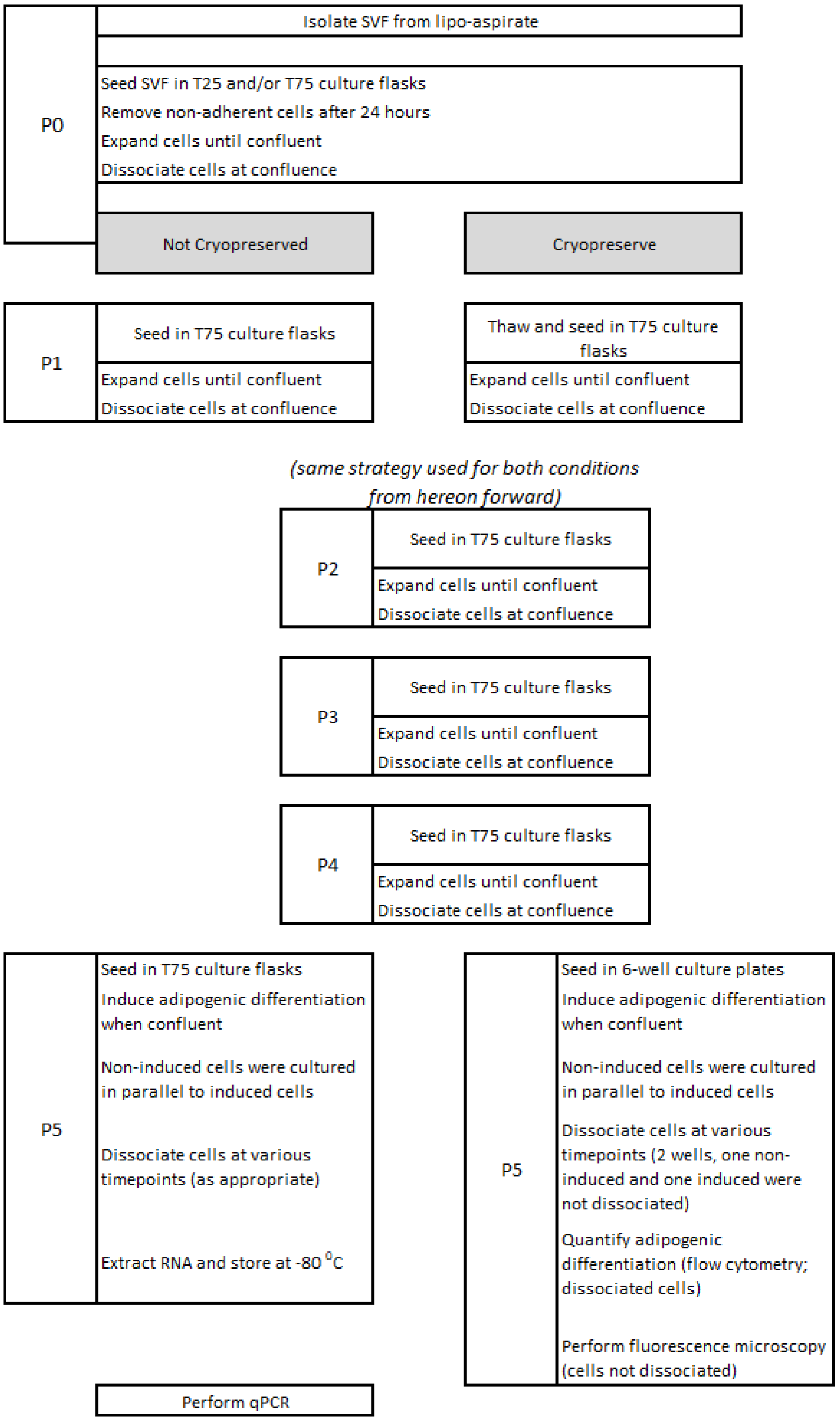

Figure S1: Experimental design to study the effect of cryopreservation on the adipogenic differentiation potential of ASCs.

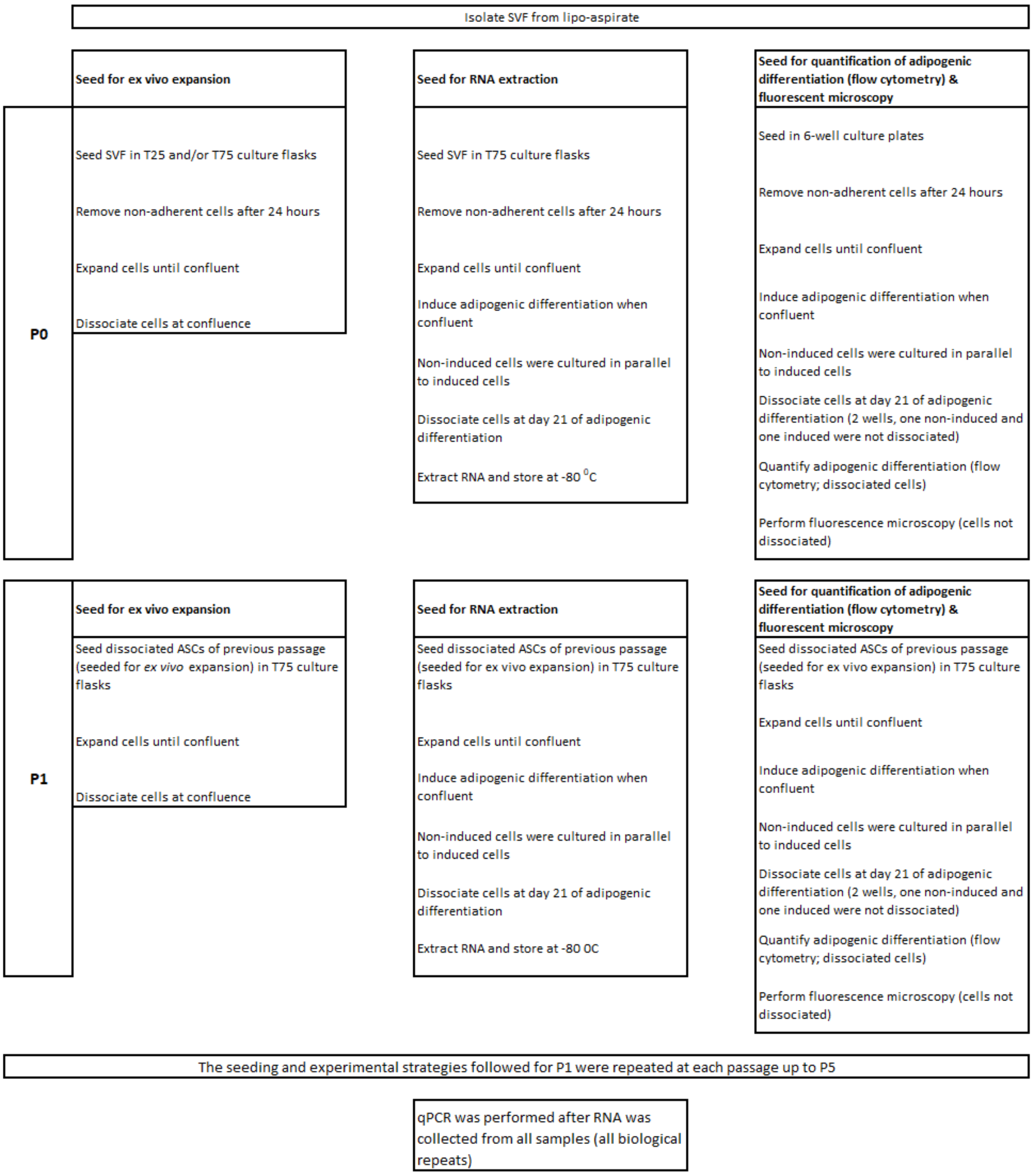

Figure S2: Experimental design to study the effect of passaging (*ex vivo* expansion) on the adipogenic differentiation potential of ASCs.

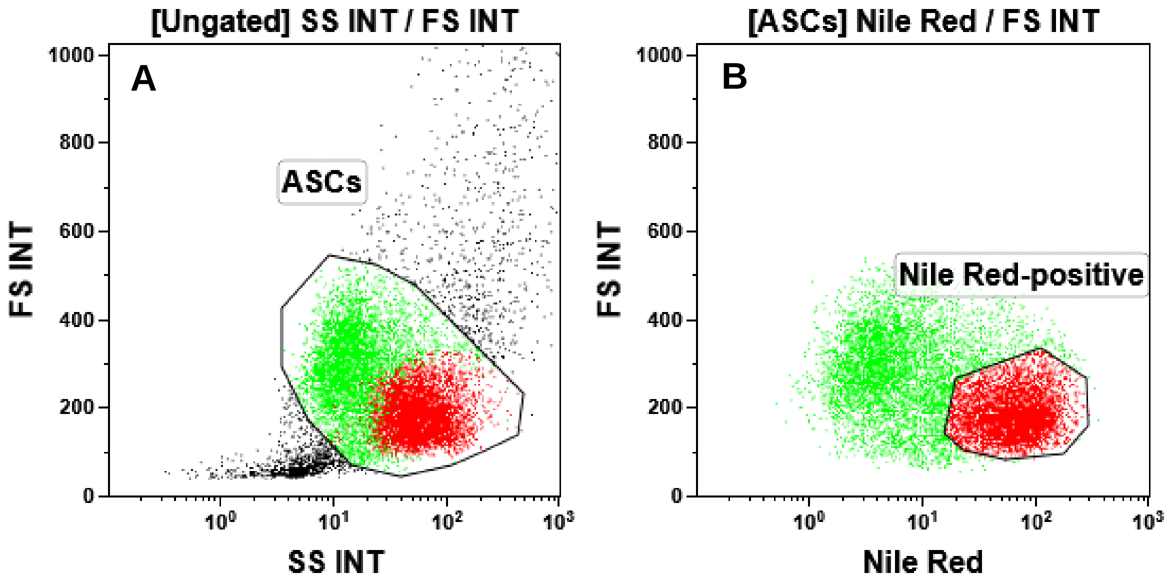

Figure S3: Representative flow cytometry data plots generated during analysis of P0 ASCs induced to differentiate into adipocytes over a 21-day differentiation period. At Day 21, the cells were stained with Nile Red to determine the proportion of cells with an increased level of intracellular lipid accumulation (B). One of the key morphological features of adipocyte differentiation is the accumulation of intracellular lipid droplets resulting in an increase in intracellular neutral lipid accumulation. Cells with increased intracellular lipid accumulation (red events in B) also display an increase in SS (red events in A). Side scatter is a flow cytometric parameter often used to differentiate between cells with different levels of intracellular complexity.

Proportion of monocytes/macrophages present in early passages of ASC cultures

Method

The proportion of monocytes/macrophages present in ASC cultures at low passages was investigated in an independent (unpublished) study using freshly isolated ASCs by staining the cells with CD14-APC Cy7 (macrophages) and CD45-Krome Orange, and the data is shown here.

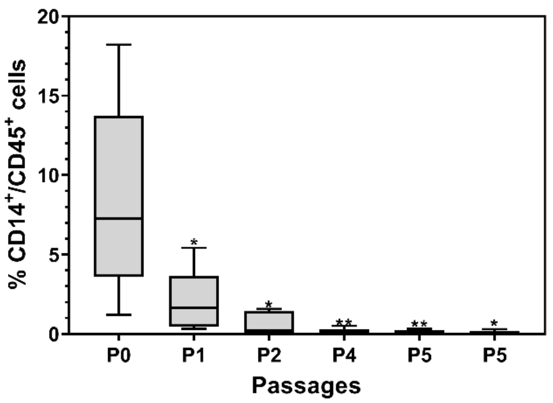

Figure S4: Percentage of CD14+ monocytes/macrophages present during *ex vivo* expansion of primary ASC cultures. Results are displayed as Tukey box-whisker plots where the median value is indicated by the solid horizontal line in each box. Results are from 6 independent ASC cultures. Significance between P0 and the other passages are indicated with an *. *$P < 0.5$; **$P < 0.01$.

Results

In a separate series of experiments, we investigated the presence of monocytes/macrophages in primary ASC cultures at the various passages indicated (P0 to P5). Monocytes/macrophages were identified as CD14+/CD45+ cells. We observed a rapid decrease in the presence of CD14+/CD45+ monocytes/macrophages in the ASC cultures with increasing rounds of *ex vivo* expansion (Supplementary Figure S2), achieving significance at P1 when compared to P0 (P = 0.041). At P0, an average of 8.42 ± 6.28% adherent cells were CD14+/CD45+, which decreased to 2.10 ± 1.94% (P = 0.04; n = 6) at P1, 0.59 ± 0.70% (P = 0.009; n = 6) at P2, 0.17 ± 0.19% (P = 0.002; n = 6) at P3, 0.14 ± 0.13% (P = 0.002; n = 6) at P4 and 0.13 ± 0.15% (P = 0.02; n = 3) at P5.
